# How Working Conditions, Socioeconomic Insecurity, and Behavior-Related Factors Mediate the Association Between Working Poverty and Health in Germany

**DOI:** 10.3389/ijph.2022.1604555

**Published:** 2022-05-11

**Authors:** Timo-Kolja Pförtner, Ibrahim Demirer

**Affiliations:** ^1^ Research Methods Division, Faculty of Human Sciences, University of Cologne, Cologne, Germany; ^2^ Institute of Medical Sociology, Health Services Research, and Rehabilitation Science, Medical Faculty and Faculty of Human Sciences, University of Cologne, Cologne, Germany

**Keywords:** employment, Germany, inequality, health, working poverty, mediation analysis

## Abstract

**Objectives:** Aims of this study were to Schmitt (Advances in Life Course Research, 2021, 47: 100402) analyze the association of working poverty with mental and physical health-related quality of life and (Wang and Ford, J Organ Behav, 2020, 41 (9): 895–914) to explain these associations by behavior-related factors (heavy drinking, smoking status, body mass index), socioeconomic insecurity (deprivation in living standards, economic worries), and mental working conditions (effort-reward imbalance, job insecurity).

**Methods:** A total of 11,500 employees aged 17–67 from the German Socioeconomic Panel (2014, 2015, and 2016) were used, and mediation analyses with inverse odds weighting stratified by gender were conducted.

**Results:** Working poverty was significantly associated with both outcomes for both genders. Deprivation in living standards contributed the most to differences in mental health, with a mediated proportion of 60.3% (men) and 44.4% (women). Differences in physical health were significantly mediated by inadequate living standards in women, with a mediated proportion of 73.7%, whereas none of the mediators considered were significant in men.

**Conclusion:** Indicators of socioeconomic insecurity contributed most to the association of working poverty with mental and physical health. Results highlight the relevance of policy initiatives to strengthen the socioeconomic living conditions of the working poor.

## Introduction

Earnings from gainful employment represent an essential factor in meeting the household’s needs and fulfilling important life opportunities. Research has shown that working poverty is associated with a low standard of living and perceived economic uncertainties that negatively impact individual life course choices and one’s own aspirations to invest in long-term goals [1, 2]. International studies from the field of public health, epidemiology, and occupational health research further indicate that employees with an income below the poverty line suffer from various detrimental health outcomes and behaviors [3], including poor self-rated health [4, 5], mental disorders [6–9], obesity [10], smoking [11, 12], alcohol consumption [13], and mortality [14].

Several hypotheses have been suggested explaining the association between working poverty and employees’ health and quality of life [3, 15]. First, a causal impact of working poverty on health has been suggested, explained by poor self-esteem and job satisfaction, deprivation in living standards and perceptions of low social status and financial uncertainties, and by lower aspirations and ability to invest in health-promoting behaviors and goods. Moreover, it is known that low-paid jobs are associated with hourly, seasonal, and temporary jobs that are characterized by instability, lack of protections, insecurity, social and economic vulnerability, and fewer opportunities for growth and benefits, which might contribute to the psychosocial burdens of the working poor [16–18]. Finally, Scott-Marshall and Tompa [19] consider unfavorable health behavior as a coping strategy concerning uncertain living and working conditions. In contrast, an opposite causal pathway between working poverty and health has also been postulated (health worker effect), which implies that healthy workers move “up” into employment with higher wages and less healthy workers move “down” into precarious employment and working poverty [20, 21]. Two systematic reviews highlight the fact that physical and mental working conditions, as well as health-related behavior play an important role in explaining health inequalities among workers [22, 23]. Due to the lack of study results, it is still unknown which further explanatory factors contribute to the association between working poverty and health.

The present study aims to investigate whether and to what extent poor mental working conditions, actual and perceived socioeconomic insecurity, and unfavorable behavior-related factors mediate the association between working poverty and mental and physical health. Data of the German Socioeconomic Panel (GSOEP) from 2014, 2015, and 2016 were used, and a mediator analysis with a weighting factor approach was applied. Analyses were stratified by gender, as associations of physical and mental health by working poverty might be stronger for men due to a violation of the traditional male breadwinner model [24]. The results obtained from this study are particularly relevant for explaining health differences among workers and provide empirical evidence on the explanation of the association between working poverty and health.

## Methods

### Study Population and Data Collection

**FIGURE 1:**
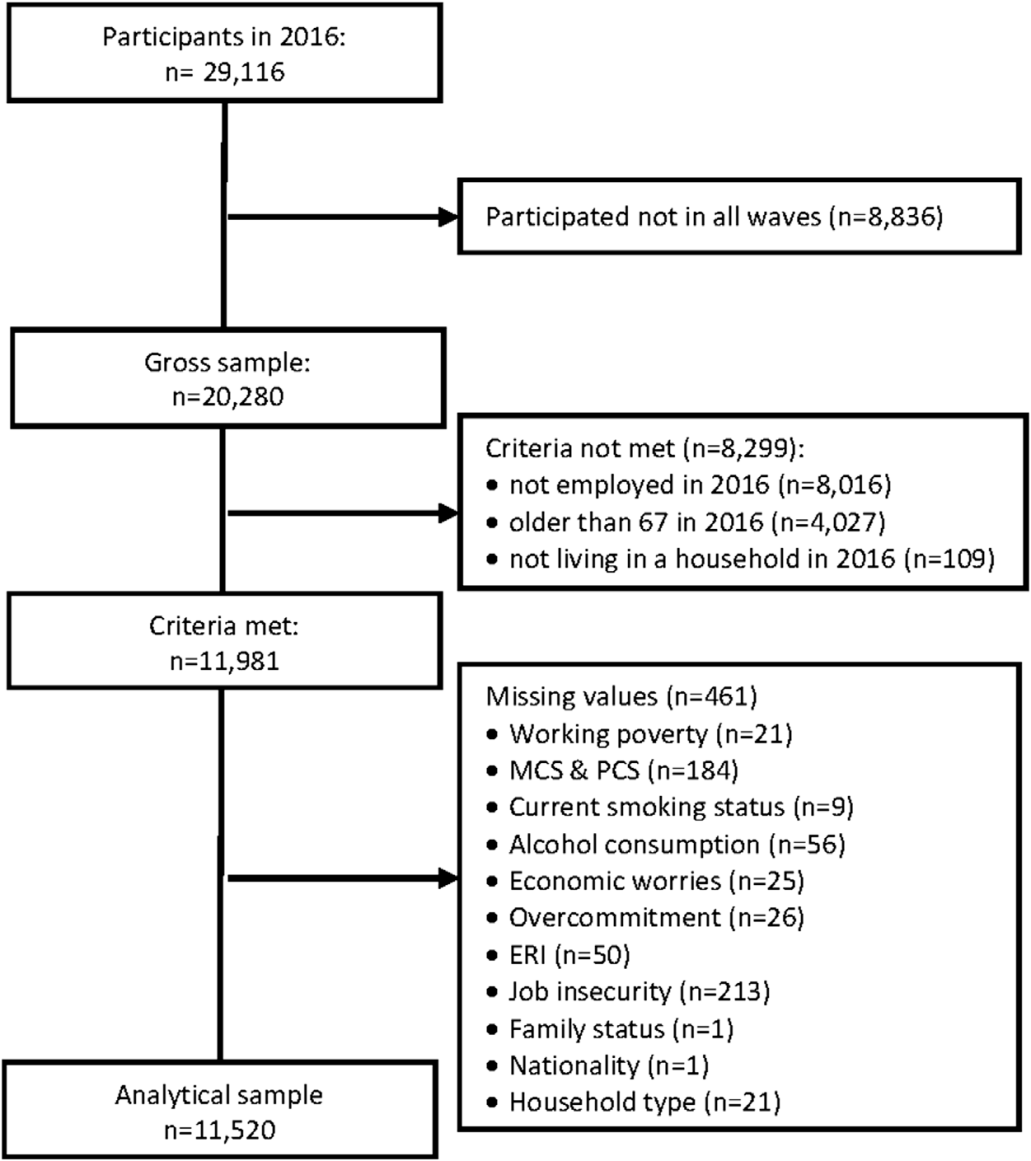
Flow chart of the analytical sample (German Socioeconomic Panel, Germany, 2014, 2015, and 2016).

The present analyses were based on the GSOEP data from 2014, 2015, and 2016 (v.34). The GSOEP is a nationally representative longitudinal household panel conducted annually by the German Institute for Economic Research since 1984 [25]. The aim of the GSOEP is to record the stability and transformation processes of living conditions in Germany. Therefore, data on demography and housing situations, personality and attitudes, education, occupation and occupational mobility, income, wealth and social security, health, worries, and satisfaction are gathered annually, interviewing all household members aged 17 years and older. In the GSOEP, households are identified through a multi-stage sampling procedure (usually by random walk) [26]. All employed respondents aged between 18 and 67 years were included for the analyses, for whom complete information on the measurement indicators used in 2014, 2015, and 2016 was available (*n* = 11,500) (see Figure 1).

### Measurements

#### Dependent Variables

We used the Physical Component Summary (PCS) and the Mental Component Summary (MCS) to indicate mental and physical health-related quality of life. The PCS and MCS were derived from the Short Form-12 Health Survey (SF-12v2), which was provided by the GSOEP in 2016. The SF-12v2 is a subset of the SF-36v2 and a multidimensional measurement tool of different aspects of health-related quality of life as physical functioning, role-physical, bodily pain, general health, vitality, social functioning, role-emotional and mental health. The SF-12-v2 thus captures various aspects of health-related quality of life in its comprehensive form. It, therefore, allows researchers to draw a far more differentiated picture of limitations in everyday life by health impairments than single-item health measures [27]. The SF-12v2 is a generally accepted and widely used tool to measure health and to detect impaired quality of life [28]. The SF-12v2 contains 12 of the original 36 items and covers 8 subscales and 2 subordinate dimensions of physical and mental health-related quality of life. Compared to the original SF-12v2, the GSOEP version deviates to some extent in the formulation and order of questions but also shows high validity [29]. The GSOEP version contains an item on “severe physical pain” instead of the item in the original SF-12v2 “work interference due to pain”. In accordance with Andersen et al. [30], the PCS and MCS are norm-based standardized (T score) to the average in mental and physical health-related quality of life in the analytical sample with a mean score set at 50 and a standard deviation of 10 ranging from 0 to 100. Therefore, coefficient effects describe deviations from the average in the analytical sample, with values above zero indicating better mental and physical health-related quality of life than the average.

#### Independent Variables

Working poverty was defined as being employed and receiving an equalized disposable household income of less than 60% of the median in the year in question [5]. In-work poverty was coded as 1 (exposed) for being working poor and coded as 0 (reference category) for being working non-poor. An alternative measure of wage income inadequacy refers to low wages that is postulated to indicate inequalities in wages at the market level and possible perceptions of deprivation [31]. We used this measure for sensitivity analyses to test for potential differences in the association of earned income at the individual and household level with physical and mental health. It was defined as gross hourly wages that are less than two-thirds of the median wage of all employees [32].

#### Mediator Variables

Five mediators were used, which refer to mental working conditions (effort-reward imbalance—ERI and perceived job insecurity), socioeconomic insecurity (deprivation in living standards and perceived economic worries), and behavior-related factors (heavy drinking, current smoking status, body mass index—BMI). Mental working conditions were indicated by the ERI and perceived job insecurity. First, the ERI measures failed reciprocity in terms of high efforts and low rewards in the workplace [33]. Among others, failed reciprocity in terms of high efforts and low rewards is more frequent in employees with excessive work-related commitment (overcommitment), which should be controlled for when using the ERI [33]. The ERI and overcommitment were measured with a short version of the ERI questionnaire in the GSOEP [34]. Effort was assessed with three items (example item: “I am often interrupted and distracted while working”), reward with seven items (example item: “My job is in jeopardy”), and overcommitment with four items (example item: “I often am already thinking about work-related problems when I wake up”) assessed with the question to what degree respondents agree with the statements (“strongly disagree,” “disagree,” “agree,” “strongly agree”). According to Siegrist et al. [35], sum scores were calculated for each scale, with higher scores reflecting higher effort, reward, and overcommitment. The ERI was calculated by dividing the effort scale score by the reward scale score (weighted by the number of items: 3/7) and set at a mean level of 1 with scores above 1 indicating failed reciprocity in terms of efforts and rewards. Second, perceived job insecurity was assessed by asking how much a person is concerned about the security of his or her job. Job insecurity was used as a binary-coded variable, distinguishing between employees with some or no concerns (value 0) and great concerns (value 1).

Socioeconomic insecurity was measured by deprivation in living standards and perceived economic worries. First, deprivation in living standards was measured by 11 activities and goods reflecting the necessary standard of living in a society [36, 37]. Respondents were asked to indicate whether the following points applied to their household and, if not, whether this was due to financial or other reasons (example item: “Furniture which is worn out but can still be used is replaced by new furniture”). Deprivation in living standards was measured using a summative index of the activities and goods missing due to financial reasons [36]. Second, perception of economic worries was used to indicate subjective feelings of socioeconomic insecurity. In the GSOEP, respondents were asked to indicate the level of concern with their own economic situation. Economic worries were dichotomized, differentiating between employees with no or some economic concerns (value 0) and employees with many economic concerns (value 1).

Behavior-related factors refer to current smoking status, heavy drinking, and BMI. For current smoking status, the GSOEP asked respondents whether they currently smoke cigarettes, a pipe, or cigars (answer categories: “No”, “Yes”). Heavy alcohol drinking was operationalized according to the definition of heavy drinkers of the Centers of Disease Control and Prevention that define a heaving drinking for women at 8 or more drinks per week and for men at 15 or more drinks per week [38]. In the GSOEP, two questionnaires related to the drinking behavior of the respondents. First, respondents were asked how often they drink alcohol (answer categories: “Every day,” “4–6 days a week”, “2–3 days a week”, “2–4 times a month”, “once a month or less frequently,” “Never”). When respondents answered that they drink alcohol, they were asked how many drinks they consumed in a day (answer categories: “1, 2 drinks”, “3, 4 drinks”, “5, 6 drinks”, “7–9 drinks”, “10 or more drinks”). A respondent was defined as a heavy drinker if the answers of both questionnaires indicated heavy drinking behavior according to the definition of the Center of Disease Control and Prevention. BMI was provided by the GSOEP and was calculated by dividing body weight in kilograms by the square of body height in meters. BMI was categorized into underweight (<18.5), normal weight (18.5–25.0), overweight (25.0–30.0), and adiposity (>30.0).

#### Covariates

The covariates from the 2014 survey wave used for the study were age, place of residence (East/West Germany), household type (one-person household/two-person household/single parent/couple with children/other), marital status (with partner or husband/no partner, divorced or widowed), nationality (German/other), and employment status of the partner (others/full-time employed).

### Statistical Analyses

The statistical analyses began with a description of the sample characteristics for the total sample, and for men and women by working poverty status. In the second step, the mediation analyses were performed. The mediation analysis was based on the assumption that working poverty must precede the mediators and the health outcomes [39]. Therefore, we used information on working poverty status from 2015 and information on the mediators and the health outcomes from 2016. Covariates from 2014 was used in order to control for potential reverse causality of the covariates with working poverty, the mediators, and the health outcomes [40].

To estimate the natural direct (NDE) and natural indirect effect (NDE) of working poverty on the health outcomes, we used the “Inverse Odds-Ratio-Weighting” method (IORW) due to advantages over traditional methods for mediation [41, 42]. First, traditional methods require correct model specifications for each regression model. The IORW method is non-parametric, requiring fewer assumptions regarding parametric distributions and model specifications. Second, examining mediation by multiple correlated mediators is often impossible or cumbersome and prone to errors in traditional methods [42]. The IORW method is not restricted to a single mediator because it does not require the coefficients of the mediators; thus, the IORW method can easily be expanded to scenarios with multiple mediators. Third, traditional parametric-based mediation methods include the mediator directly in the model estimation, which could cause a violation of the no exposure-mediator interaction assumption. The IORW method does not make any assumption of the exposure-mediator interaction.

The IORW method is a non-parametric approach to mediation within a counterfactual framework. The IORW method circumvents problems of traditional models by using inverse probability weights (IPW) instead of estimating actual paths when identifying the NDE. These weights condense the information on the odds ratio (OR) between exposure and mediators, conditional on the covariates, and estimate the NDE via weighted regression analysis [41]. The method takes advantage of the invariance property of odds ratios, which results in the same OR between two variables, regardless of which is considered the dependent variable. Therefore, an OR for an exposure as a function of a mediator is equivalent to an OR for a mediator as a function of the exposure. The invariance property also applies to multiple mediators, so that a regression does not have to be calculated for each mediator, but a regression of the exposure on all mediators is used to estimate the IPW [41]. These weights are entered into a weighted regression to calculate the NDE. Therefore, the mediators are never considered in the regression model for the outcome and are only used in the construction of the weight [42]. To obtain the NIE, the IORW method uses the difference method by subtracting the NDE (derived from a weighted regression model with the IPW) from the TE (derived from a non-weighted regression model without the IPW) [43].

The calculation of IPWs–shown here, exemplary for all mediators–was based on a logistic regression model to predict working poverty $\hat{P}\left(WP\right)$ .

$$\hat{P}\left(WP\right)=logit\;\hat{P}\text{\{}WP\left(2015\right)\text{|}\;Behav\left(2016\right),EconInsec\left(2016\right),\;EconWor\left(2016\right),\;MentWork\left(2016\right),\;C\left(2014/2016\right)\}$$

The model included the mediators current smoking status, heavy drinkers, and BMI (*Behav*), deprivation at the household level and economic worries (*EconInsec*), and effort-reward imbalance and job insecurity (*MentWork*). Covariates (*C*) from 2014 and overcommitment from 2016 were included to consider the temporal influence of these factors on working poverty. The predicted values [ $\hat{P}\left(WP\right)$ ] were assigned as inverse weights to the working poor ( WP=1) . Non-working poor ( WP=0) received the reference value 1:

$$IPW\left(WP=1\right)=\frac{1-\hat{P}\left(WP\right)}{\hat{P}\left(WP\right)};\;IPW\left(WP=0\right)=1$$

The estimation of the TE and the NDE was based on a generalized linear model for Gaussian-distributed outcome variables using the power-link function. All models included the control variables from the 2014 survey wave. Following the IORW approach, the estimation of the TE did not apply IPWs. Only in the estimation of the NDE, was the IPW in question used. The confidence intervals of the TE, NDE, and NIE were estimated using the bootstrap-resampling method from 1,000 samples of the present dataset [44]. The mediating effect was determined for all mediators combined, for all behavior-related factors, indicators of socioeconomic insecurity, and mental working conditions combined, and for each mediator separately. The proportion mediated (PM) captures how much of the effect of working poverty on mental and physical health is due to the effect of working poverty on the intermediate. The PM is calculated by dividing the NIE by the TE. Sensitivity analyses were conducted using low income as the exposure variable to check the robustness of our findings. All analyses were performed with Stata V16.0 (Stata Corp, College Station, TX, United States).

## Results

Table 1 presents descriptive information on the analytical sample. Among the employed participants in the GSOEP, 11.3% of men and 14.4% of women experienced working poverty. The working poor were more often younger, from East Germany, single parents, employees with no partner, and employees living in households with no full-time employed partner. The prevalence of working poverty was more pronounced for women in particular with regard to the household and family type, and the employment status of the partner. Mean levels of mental and physical health-related quality of life were slightly lower for male and female working poor. Current smoking status was more prevalent among the working poor, whereas alcohol use was more prevalent among the non-working poor. Compared to the non-working poor, adiposity was more common among the working poor. The working poor showed higher mean levels of deprivation, perceptions of economic worries, and were more often very concerned about their job security, whereas the ERI and overcommitment did not strongly differ between the non- and the working poor.

**TABLE 1:** Sample characteristics of employed individuals (German Socioeconomic Panel, Germany, 2014, 2015, and 2016, *n* = 11,469).

	Total (*n* = 11,520)	Men (n = 5,466)		Women (n = 6,054)	
		Non-working poor	Working poor	Non-working poor	Working poor
Total	100.0	88.7	11.3	85.6	14.4
**Control variables**					
Age (%)					
17–29	12.5	11.5	21.9	11.0	19.9
30–39	24.1	23.2	23.2	25.1	23.8
40–49	33.3	33.4	30.8	34.1	30.9
50–67	30.0	31.9	24.0	29.8	25.4
Place of residence					
East Germany (%)	22.1	21.4	25.3	21.6	25.7
West Germany	77.9	78.6	74.7	78.4	74.1
Nationality (%)					
German	90.2	91.2	76.8	91.8	85.1
Other	9.8	8.8	23.2	8.2	14.9
Household type (%)					
One-Person-HH	10.3	11.1	11.8	9.2	11.9
Two-Person-HH	21.4	23.3	7.6	23.5	8.5
Single parent	9.2	2.8	6.8	11.6	31.6
Couple with Children	56.9	60.8	68.7	53.9	44.3
Other	2.2	2.0	5.2	1.8	3.7
Family type (%)					
With partner/husband	81.1	84.8	76.8	81.5	61.3
No partner, divorced, widowed	18.9	15.2	23.2	18.5	38.7
Employment status of partner (%)					
Others	63.2	77.7	92.4	44.3	74.7
Full-Time employed	36.8	22.3	7.7	55.7	25.3
Overcommitment (mean)	12.6	12.7	12.2	12.6	11.6
**Dependent variables**					
Mental health-related quality of life (mean)	50.0	51.2	50.7	49.1	48.0
Physical health-related quality of life (mean)	50.0	50.8	49.6	49.7	47.5
**Mediator variables**					
Current smoking status (%)					
Not smoking	72.1	69.9	57.4	76.5	67.8
Smoking	27.9	30.1	42.6	23.5	32.2
Heavy drinker (%)					
No	90.0	90.7	93.6	88.5	91.8
Yes	10.0	9.3	6.4	11.5	8.2
BMI (%)					
Underweight (<18.5)	1.3	0.2	1.1	2.1	2.4
Normal weight (18.5–25.0)	43.3	33.1	37.3	53.2	44.9
Overweight (25.0–30.0)	35.4	45.2	40.6	26.9	27.7
Adiposity (>30.0)	20.0	21.4	21.0	17.8	24.9
Deprivation in household (mean)	0.8	0.6	2.3	0.6	2.4
Perceptions of economic worries (%)					
Not/somewhat concerned	87.2	89.5	79.8	88.2	73.9
Very concerned	12.8	10.5	20.2	11.8	26.1
Effort-reward imbalance (mean)	1.0	1.0	1.0	1.0	0.9
Perceived job insecurity (%)					
Some/no concerned	93.0	93.6	84.8	94.2	88.3
Very concerned	7.0	6.4	15.2	5.8	11.7

Table 2 shows differences in mental health-related quality of life by working poverty status and the impact of the mediators for men and women. For men and women, the TE implies a significant association between working poverty and mental health-related quality of life for men by −0.999 (95% CI: −1.791; −0.208) and for women by -1.406 (95% CI: −2.216; −0.596). Therefore, male working poor deviated from the average in mental health-related quality of life in the analytical sample by −0.999 and female working poor by −1.406. The consideration of all mediators was associated with a non-significant NIE of -0.335 (95% CI: −1.270; 0.600) for men and a significant NIE of −1.147 (95% CI: −2.005; −0.290) for women, and implied a full mediation process resulting in an NDE of −0.665 (95% CI: −1.895; 0.566) for men and an NDE of −0.258 (95% CI: −1.406; 0.889) for women. A positive non-significant NIE was observed for heavy drinking, smoking, BMI, the ERI, and job insecurity for both genders. For women, the consideration of indicators of socioeconomic insecurity was associated with the strongest reduction in differences in mental health-related quality of life status by working poverty status. In contrast, for men, only the consideration of economic worries reduced differences in mental health-related quality of life by working poverty status. For women, the reduction of differences in mental health by working poverty status was the strongest for deprivation in living standards with a mediated proportion of 84.5%, and for men for economic worries with a mediated proportion of 5.9%.

**TABLE 2:** Total effect (TE), natural direct effect (NDE), and natural indirect effect (NIE) of working poverty on mental health-related quality of life for men and women considering heavy drinking, current smoking status, BMI, deprivation in living standards, economic worries, ERI, and job insecurity as mediators (German Socioeconomic Panel, Germany, 2014, 2015, and 2016).

	Men (*n* = 5,466)				Women (*n* = 6,054)			
	β	95%-CI	p-value	PM in %	β	95%-CI	p-value	PM in %
All mediators								
NIE	−0.335	−1.270; 0.600	0.483	33.5%	−1.147	−2.005; −0.290	0.009	81.6%
NDE	−0.665	−1.895; 0.566	0.290		−0.258	−1.406; 0.889	0.659	
Health behaviors (all)								
NIE	0.240	−0.508; 0.988	0.529	−24.0%	0.058	−0.651; 0.767	0.873	−4.1%
NDE	−1.239	−2.406; 0.988	0.037		−1.464	−2.542; −0.386	0.008	
Heavy drinking								
NIE	0.364	−0.337; 1.065	0.308	−36.4%	0.307	−0.407; 1.020	0.400	−21.8%
NDE	−1.363	−2.474; −0.253	0.016		−1.713	−2.793; −0.632	0.002	
Smoking								
NIE	0.306	−0.472; 1.083	0.441	−30.5%	0.219	−0.477; 0.915	0.538	−15.6%
NDE	−1.305	−2.485; −0.125	0.030		−1.625	−2.697; −0.552	0.003	
BMI								
NIE	0.317	−0.405; 1.039	0.389	−31.7%	0.105	−0.629; 0.838	0.780	−7.5%
NDE	−1.316	−2.396; −0.237	0.017		−1.511	−2.558; −0.463	0.005	
Socioeconomic insecurity (all)								
NIE	0.006	−0.903; 0.915	0.990	−0.5%	−1.188	−2.097; −0.278	0.010	84.5%
NDE	−1.005	−2.199; 0.188	0.099		−0.218	−1.364; 0.927	0.709	
Deprivation in living standards								
NIE	0.034	−0.867; 0.934	0.941	−3.4%	−1.036	−2.000; −0.071	0.035	73.7%
NDE	−1.033	−2.239; 0.172	0.093		−0.370	−1.593; 0.853	0.553	
Economic worries								
NIE	−0.059	−0.796; 0.678	0.876	5.9%	−0.495	−1.213; 0.224	0.177	35.2%
NDE	−0.940	−2.044; 0.164	0.095		−0.911	−1.952; 0.129	0.086	
Mental working conditions (all)								
NIE	0.063	−0.705; 0.831	0.872	−6.3%	0.198	−0.594; 0.991	0.623	−14.1%
NDE	−1.062	−2.249; 0.124	0.079		−1.604	−2.754; −0.455	0.006	
Effort-reward imbalance								
NIE	0.227	−0.521; 0.975	0.552	−22.7%	0.392	−0.367; 1.152	0.311	−27.9%
NDE	−1.226	−2.364; −0.088	0.035		−1.798	−2.894; −0.703	0.001	
Job insecurity								
NIE	0.146	−0.640; 0.933	0.715	−14.6%	0.124	−0.627; 0.875	0.747	−8.8%
NDE	−1.146	−2.315; 0.024	0.055		−1.530	−2.658; −0.402	0.008	
TE	−0.999	−1.791; −0.208	0.013		−1.406	−2.216; −0.596	0.001	

Notes: all models were adjusted for age, place of residence, household type, marital status, and nationality from the 2014 survey wave and for overcommitment from the 2016 wave. PM: proportion mediated.

Table 3 shows the differences in physical health-related quality of life by working poverty status and the impact of the mediators for both genders. The TE reveals that working poverty was significantly associated with a lower physical health-related quality of life of -2.164 (95% CI: −2.952; −1.375) for men, and of −2.869 (95% CI: −3.644; −2.094) for women. The consideration of all mediators was associated with a significant NIE of −1.369 (95% CI: −2.211; −0.528) for men and a significant NIE of −1.531 (95% CI: −2.402; −0.659) for women, and implied a full mediation process resulting in an NDE of −0.795 (95% CI: −1.905; 0.316) for men and an NDE of −1.338 (95% CI: −2.481; −0.195) for women. Among all mediators, the strongest reduction in differences in physical health-related quality of life was observed for deprivation in living standards with a proportion mediated of 60.3% for men, and 44.4% for women. The results revealed a positive NIE for heavy drinking, smoking, ERI, and job insecurity for both genders, and for BMI but only for men.

**TABLE 3:** Total effect (TE), natural direct effect (NDE), and natural indirect effect (NIE) of working poverty on physical health-related quality of life for men and women considering heavy drinking, current smoking status, BMI, deprivation in living standards, economic worries, ERI, and job insecurity as mediators (German Socioeconomic Panel, Germany, 2014, 2015, and 2016).

	Men (*n* = 5,466)					Women (*n* = 6,054)			
	β	95%-CI	p-value	PM in %	β		95%-CI	p-value	PM in %
All mediators									
NIE	−1.369	−2.211; −0.528	0.001	63.3%	−1.531		−2.402; −0.659	0.001	53.4%
NDE	−0.795	−1.905; 0.316	0.161		−1.338		−2.481; −0.195	0.022	
Health behaviors (all)									
NIE	0.035	−0.563; 0.633	0.909	−1.6%	−0.597		−1.369; 0.176	0.130	20.8%
NDE	−2.198	−3.142; −1.255	<0.001		−2.272		−3.357; −1.187	<0.001	
Heavy drinking									
NIE	0.363	−0.258; 0.983	0.252	−16.8%	0.080		−0.612; 0.772	0.822	−2.8%
NDE	−2.527	−3.552; −1.502	<0.001		−2.948		−3.987; −1.909	<0.001	
Smoking									
NIE	0.077	−0.527; 0.681	0.803	-3.5%	0.057		−0.626; 0.740	0.870	−2.0%
NDE	−2.241	−3.233; −1.248	<0.001		−2.925		−3.967; −1.884	<0.001	
BMI									
NIE	0.350	−0.288; 0.988	0.282	−16.1%	−0.460		−1.111; 0.250	0.216	16.0%
NDE	−2.514	−3.549; −1.479	<0.001		−2.438		−3.501; −1.376	<0.001	
Socioeconomic insecurity (all)									
NIE	−1.356	−2.181; −0.531	0.001	62.7%	−1.341		−2.200; −0.482	0.002	46.7%
NDE	−0.808	−1.923; 0.306	0.156		−1.523		−2.644; −0.411	0.007	
Deprivation in living standards									
NIE	−1.304	−2.143; −0.466	0.002	60.3%	−1.273		−2.154; −0.393	0.005	44.4%
NDE	−0.859	−d2.042; 0.324	0.154		−1.595		−2.762; −0.428	0.007	
Economic worries									
NIE	0.043	−0.634; 0.719	0.902	−2.0%	−0.251		−0.990; 0.489	0.506	8.7%
NDE	−2.206	−3.220; −1.191	<0.001		−2.618		−3.681; −1.555	<0.001	
Mental working conditions (all)									
NIE	0.097	−0.582; 0.775	0.780	−2.3%	0.148		−0.581; 0.877	0.691	−5.1%
NDE	−2.260	−3.315; −1.206	<0.001		−3.016		−4.073; −1.959	<0.001	
Effort-reward imbalance									
NIE	0.235	−0.400; 0.871	0.468	−10.9%	0.317		−0.374; 1.009	0.368	−11.1%
NDE	−2.399	−3.473; −1.325	<0.001		−3.186		−4.217; −2.155	<0.001	
Job insecurity									
NIE	0.163	−0.495; 0.821	0.627	−7.5%	0.017		−0.656; 0.690	0.961	−0.6%
NDE	−2.327	−3.296; −1.358	<0.001		−2.886		−3.915; −1.856	<0.001	
TE	−2.164	−2.952; −1.375	<0.001		−2.869		−3.644; −2.094	<0.001	

Notes: all models were adjusted for age, place of residence, household type, marital status, and nationality from the 2014 survey wave and for overcommitment from the 2016 wave. PM: proportion mediated.

Sensitivity analysis with low income as an indicator of wage income adequacy generally differs only slightly from the results on working poverty (results presented in Supplementary Tables S1, S2). The TE in the association of low income with mental and physical health-related quality of life was weaker compared to the TE of working poverty. Again, the consideration of indicators of socioeconomic insecurity was associated with the strongest reduction in differences in mental health-related quality of life for both genders.

## Discussion

This is the first study analyzing whether and to what extent mental working conditions, socioeconomic insecurity, and behavior-related factors mediate the association between working poverty and mental and physical health. Using a novel mediation approach, the results showed that socioeconomic insecurity and deprivation in living standards, in particular, contributed the most to the differences in mental and physical health by working poverty status. Behavior-related factors and mental working conditions only slightly contribute to explaining the differences in physical and mental health-related quality of life by working poverty status. In particular, ERI–as part of the mental working conditions–was indicated to be an inverse mediator of the association between working poverty and mental and physical health-related quality of life. The results did not strongly differ by gender and when low wage was used as the exposure variable.

This study found that indicators of socioeconomic insecurity and deprivation in living standards contribute largely to the association between working poverty and mental and physical health-related quality of life. Indicators of socioeconomic insecurity are strongly related to financial strain, which has been shown to mediate the association between precarious employment and mental health [45]. The strong relevance of deprivation in living standards for differences in mental and physical health-related quality of life by working poverty status might be explained by the substantial burden of deprivation in living standards. A recent study has shown that deprivation in living standards was the main contributor to income-related inequalities in health besides feelings of financial worries [46]. Deprivation includes not only disadvantages in material living standards, but also a feeling of social exclusion [47]. This has been impressively demonstrated in the qualitative study by Premji [48]. In this study, people with low income wages spoke about being unable to afford clothes, shoes, and food, and limited or prevented social activities due to their economic insecurity. Moreover, several authors highlighted that deprivation in living standards results from a prolonged spell of insufficient income when financial savings are exhausted [37]. Therefore, deprivation might also imply a deep and long-lasting socioeconomic disadvantage over the life course, which contributes to the hypothesis that the working poor differ strongly in their socioeconomic and health situation from other employees [5, 49].

The behavior-related mediators could not explain differences in health-related quality of life by working poverty. A recent study of Schram et al. [50] could also not find a contribution of current smoking status, alcohol consumption, and BMI explaining educational inequalities in SRH among employees. A reason might be that other factors, such as deprivation in living standards and economic worries, have a more substantial impact on mental and physical health for the working poor than behavior-related factors. A similar explanation might be valid for the findings on the relevance of the ERI explaining the association between working poverty and physical and mental health-related quality of life. In line with our study, a review of Hoven and Siegrist [23] confirmed that socioeconomically disadvantaged employees are more often exposed to an ERI but found no or little contribution of an ERI explaining socioeconomic inequalities in health. A reason for this might be that the socioeconomic living conditions play a more critical role for the health of the working poor than the mental working conditions. Moreover, according to Standing, precarious employment is associated “with a habituation to expecting a life of unstable labor and unstable living” [51]. Therefore, it might be possible that the working poor, as opposed to other employees, are familiar with an imbalance between effort and reward at work.

### Strengths and Limitations of This Study

Strengths of the study include its large representative sample of employees, consistency in sampling and measurement over two panel waves, and multiple mediators. Also, the use of mental and physical health indicators provided a more complete account of differences in health than that either could provide as a single health measure. Additionally, the novel approach of mediator analyses allowed us to avoid violation of the no exposure-mediator interaction.

However, the study has some limitations. First, results indicated significant but rather small differences in physical and mental health by working poverty status, especially in comparison to studies with a single-item health outcome [5, 49]. Various aspects may have contributed to this. Inequalities in health may be underestimated due to an attrition and selection bias of unhealthy employees with a low income [50]. In addition, some working poor may have adapted to their health situation, and attenuation bias due to measurement error might have further contributed to underestimating differences in physical and mental health by working poverty [50]. Finally, the scaling and the depth of meaning of the various health indicators have an influence on the results and the differences to other studies that used a single-item health outcome. While single-item health outcomes are often used as a binary variable, measuring in particular general health and thus mixing essential aspects of mental and physical health, the MCS and PCS provide a more detailed and fine-grained insight into health. Thus, while the SRH provides a rather crude view of people’s health and separates the health status of individuals into poor and good, the MCS and PCS capture health on a continuum that does not emphasize differences in health as much as single-item health outcomes. Second, the relevance of working conditions might be underestimated, as we had only information about the ERI and perceptions of job insecurity. Studies have found that the physical and other mental working conditions, such as the physical and chemical hazards or job-demand control and social support at work, contribute to inequalities in health among employees [23]. Third, according to Schram et al. [50], our study might also be biased due to unmeasured confounders, such as personality, ability, genetics, or childhood circumstances, which may have affected differences in physical and mental health, and the mediators. This might be particularly valid for differences in physical health, which strongly depended on health conditions from the previous survey wave, indicating a polarization in health by working poverty status over the life course [5]. Fourth, employment status and wage income were measured only once annually, although transitions in employment and wage income might have taken place within the survey year.

### Conclusion

In conclusion, our study shows that indicators of socioeconomic insecurity and deprivation in living standards strongly contributed to the association between working poverty and mental and physical health. The study results suggest the relevance of social policy initiatives to strengthen the socio-economic living conditions of the working poor.
