# Supplementary material for: How Working Conditions, Socioeconomic Insecurity, and Behavior-Related Factors Mediate the Association Between Working Poverty and Health in Germany
Source: Int J Public Health. 2022 May 11;67:1604555. doi: 10.3389/ijph.2022.1604555 (PMC9130479; doi:10.3389/ijph.2022.1604555)
Supplement: Supplementary file 1 [file DataSheet1.docx]

**Supplementary Table 1**: Total effect (TE), natural direct effect (NDE), and natural indirect effect (NIE) of low income on mental health-related quality of life for men and women considering heavy drinking, current smoking status, BMI, deprivation in living standards, economic worries, ERI, and job insecurity as mediators (German Socioeconomic Panel 2014, 2015 & 2016).

|  | Men (n=5,466) | | | | | Women (n=6,054) | | | |
| --- | --- | --- | --- | --- | --- | --- | --- | --- | --- |
|  | | β | 95%-CI | p-value | PM in % | β | 95%-CI | p-value | PM in % |
| All mediators | | |  |  |  |  |  |  |  |
| NIE | | 0.103 | -0.494; 0.699 | 0.736 | -11.1% | -0.578 | -0.817; -0.339 | <0.001 | 110.9% |
| NDE | | -1.024 | -1.968; -0.080 | 0.034 |  | 0.056 | -0.545; 0.659 | 0.854 |  |
| Health behaviors (all) | | |  |  |  |  |  |  |  |
| NIE | | 0.402 | -0.114; 0.917 | 0.127 | -43.6% | -0.086 | -0.281; 0.108 | 0.384 | 16.5% |
| NDE | | -1.323 | -2.250; -0.395 | 0.005 |  | -0.435 | -1.041; 0.171 | 0.159 |  |
| Heavy drinking | | |  |  |  |  |  |  |  |
| NIE | | 0.405 | -0.041; 0.852 | 0.075 | -44.0% | 0.022 | -0.128; 0.172 | 0.773 | -4.2% |
| NDE | | -1.327 | -2.211; 0.442 | 0.003 |  | -0.543 | -1.173; 0.087 | 0.091 |  |
| Smoking | |  |  |  |  |  |  |  |  |
| NIE | | 0.330 | -0.166; 0.827 | 0.192 | -35.8% | -0.065 | -0.228; 0.098 | 0.433 | 12.5% |
| NDE | | -1.252 | -2.177; -0.326 | 0.008 |  | -0.456 | -1.076; 0.164 | 0.149 |  |
| BMI | |  |  |  |  |  |  |  |  |
| NIE | | 0.436 | -0.018; 0.889 | 0.060 | -47.3% | -0.080 | -0.258; 0.099 | 0.381 | 15.3% |
| NDE | | -1.357 | -2.251; -0.463 | 0.003 |  | -0.442 | -1.046; 0.163 | 0.152 |  |
| Socioeconomic insecurity (all) | | | |  |  |  |  |  |  |
| NIE | | 0.009 | -0.496; 0.514 | 0.972 | -1.0% | -0.631 | -0.865; -0.397 | <0.001 | 121.1% |
| NDE | | -0.930 | -1.800; -0.060 | 0.036 |  | 0.110 | -0.503; 0.723 | 0.725 |  |
| Deprivation in living standards | | | |  |  |  |  |  |  |
| NIE | | 0.084 | -0.462; 0.629 | 0.763 | 9.0% | -0.503 | -0.732; -0.274 | <0.001 | 96.5% |
| NDE | | -1.005 | -1.941; -0.069 | 0.035 |  | -0.019 | -0.627; 0.590 | 0.953 |  |
| Economic worries | | |  |  |  |  |  |  |  |
| NIE | | -0.083 | -0.495; 0.329 | 0.692 | 9.0% | -0.380 | -0.549; -0.210 | <0.001 | 72.9% |
| NDE | | -0.838 | -1.668; -0.009 | 0.048 |  | -0.142 | -0.734; 0.451 | <0.001 |  |
| Mental working conditions (all) | | |  |  |  |  |  |  |  |
| NIE | | 0.331 | -0.140; 0.802 | 0.168 | -35.9% | -0.088 | -0.244; 0.068 | 0.271 | 16.9% |
| NDE | | -1.252 | -2.148; -0.357 | 0.006 |  | -0.433 | -1.048; 0.181 | 0.167 |  |
| Effort-reward imbalance | | |  |  |  |  |  |  |  |
| NIE | | 0.367 | -0.090; 0.825 | 0.116 | -36.7% | 0.011 | -0.141; 0.164 | 0.884 | -2.1% |
| NDE | | -1.289 | -2.191; -0.387 | 0.005 |  | -0.533 | -1.133; 0.068 | 0.082 |  |
| Job insecurity | | |  |  |  |  |  |  |  |
| NIE | | 0.332 | -0.134; 0.799 | 0.163 | -36.0% | -0.122 | -0.279; 0.036 | 0.130 | 23.4% |
| NDE | | -1.254 | -2.196; -0.312 | 0.009 |  | -0.400 | -1.018; 0.219 | 0.205 |  |
| TE | | -0.921 | -1.638; -0.205 | 0.012 |  | -0.521 | -1.0802; 0.038 | 0.063 |  |
| Notes: all models were adjusted for age, place of residence, household type, marital status, and nationality from the 2014 survey wave and for overcommitment from the 2016 wave. PM: Proportion mediated. | | | | | | | | | |

**Supplementary Table 2**: Total effect (TE), natural direct effect (NDE), and natural indirect effect (NIE) of low income on physical health-related quality of life for men and women considering heavy drinking, current smoking status, BMI, deprivation in living standards, economic worries, ERI, and job insecurity as mediators (German Socioeconomic Panel 2014, 2015 & 2016).

|  | Men (n=5,466) | | | | Women (n=6,054) | | | |
| --- | --- | --- | --- | --- | --- | --- | --- | --- |
|  | β | 95%-CI | p-value | PM in % | β | 95%-CI | p-value | PM in % |
| All mediators | |  |  |  |  |  |  |  |
| NIE | -0.461 | -1.036; 0.115 | 0.117 | 34.7% | -0.835 | 1.088; -0.583 | <0.001 | 41.2% |
| NDE | -0.662 | -1.559; 0.236 | 0.148 |  | -1.154 | -1.786; -0.521 | <0.001 |  |
| Health behaviors (all) | |  |  |  |  |  |  |  |
| NIE | 0.204 | -0.259; 0.668 | 0.387 | -15.4% | -0.512 | -0.705; -0.319 | <0.001 | 25.7% |
| NDE | -1.327 | -2.186; -0.468 | 0.002 |  | -1.477 | -2.088; -0.867 | <0.001 |  |
| Heavy drinking | |  |  |  |  |  |  |  |
| NIE | 0.356 | -0.079; 0.791 | 0.108 | -20.7% | 0.025 | -0.126; 0.177 | 0.743 | -1.2% |
| NDE | -1.479 | -2.339; -0.619 | 0.001 |  | -2.014 | -2.606; -1.423 | <0.001 |  |
| Smoking |  |  |  |  |  |  |  |  |
| NIE | 0.233 | -0.242; 0.707 | 0.336 | -20.6% | -0.089 | -0.261; 0.084 | 0.313 | 4.5% |
| NDE | -1.355 | -2.234; -0.477 | 0.003 |  | -1.900 | -2.494; -1.307 | <0.001 |  |
| BMI |  |  |  |  |  |  |  |  |
| NIE | 0.379 | -0.070; 0.828 | 0.098 | -33.8% | -0.347 | -0.515; -0.179 | <0.001 | 17.4% |
| NDE | -1.501 | -2.394; -0.609 | 0.001 |  | -1.642 | -2.269; -1.015 | <0.001 |  |
| Socioeconomic insecurity (all) | | |  |  |  |  |  |  |
| NIE | 0.532 | -1.091; 0.028 | 0.063 | 47.4% | -0.486 | -0.708; -0.263 | <0.001 | 24.4% |
| NDE | -0.591 | -1.477; 0.296 | 0.192 |  | -1.504 | -2.094; -0.913 | <0.001 |  |
| Deprivation in living standards | | |  |  |  |  |  |  |
| NIE | -0.494 | -1.040; 0.051 | 0.076 | 44.0% | -0.397 | -0.620; -0.174 | <0.001 | 20.0% |
| NDE | -0.628 | -1.563; 0.307 | 0.188 |  | -1.592 | -2.205; -0.980 | <0.001 |  |
| Economic worries | |  |  |  |  |  |  |  |
| NIE | 0.041 | -0.404; 0.486 | 0.857 | -3.6% | -0.191 | -0.374; -0.009 | 0.040 | 9.6% |
| NDE | -1.164 | -2.044; -0.283 | 0.001 |  | 1.798 | -2.413; -1.182 | <0.001 |  |
| Mental working conditions (all) | |  |  |  |  |  |  |  |
| NIE | 0.276 | -0.175; 0.728 | 0.231 | -24.6% | -0.014 | -0.187; 0.159 | 0.873 | 0.7% |
| NDE | -1.399 | -2.259; -0.538 | 0.001 |  | -1.975 | -2.582; -1.368 | <0.001 |  |
| Effort-reward imbalance | |  |  |  |  |  |  |  |
| NIE | 0.332 | -0.122; 0.787 | 0.152 | -29.6% | 0.077 | -0.080; 0.234 | 0.399 | -3.9% |
| NDE | -1.455 | -2.341; -0.569 | 0.001 |  | -2.066 | -2.665; -1.466 | <0.001 |  |
| Job insecurity | |  |  |  |  |  |  |  |
| NIE | 0.279 | -0.184; 0.742 | 0.238 | -24.8% | -0.025 | -0.195; 0.144 | 0.768 | 1.3% |
| NDE | -1.401 | -2.306; -0.497 | 0.002 |  | -1.964 | -2.567; -1.361 | <0.001 |  |
| TE | -1.123 | -1.834; -0.411 | 0.002 |  | -1.989 | -2.569; -1.410 | <0.001 |  |
| Notes: all models were adjusted for age, place of residence, household type, marital status, and nationality from the 2014 survey wave and for overcommitment from the 2016 wave. PM: Proportion mediated. | | | | | | | | |
